# Near Field Enhanced Photocurrent Generation in P-type Dye-Sensitized Solar Cells

**DOI:** 10.1038/srep03961

**Published:** 2014-02-04

**Authors:** Xiaobao Xu, Jin Cui, Junbo Han, Junpei Zhang, Yibo Zhang, Lin Luan, Getachew Alemu, Zhong Wang, Yan Shen, Dehua Xiong, Wei Chen, Zhanhua Wei, Shihe Yang, Bin Hu, Yibing Cheng, Mingkui Wang

**Affiliations:** 1 Michael Grätzel Center for Mesoscopic Solar Cells, Wuhan National Laboratory for Optoelectronics, School of Optical and Electronic Information, Huazhong University of Science and Technology, 1037 Luoyu Road, 430074 Wuhan, People's Republic China; 2 Wuhan National High Magnetic Field Center, School of Physics, Huazhong University of Science and Technology, 1037 Luoyu Road, 430074 Wuhan, People's Republic China; 3 Department of Chemistry, The Hong Kong University of Science and Technology, Clear Water Bay, Kowloon, Hong Kong, People's Republic China; 4 Department of Materials Science and Engineering, University of Tennessee, Knoxville, TN 37996-2100; 5 Department of Materials Engineering, Monash University, Melbourne, Victoria, 3800, Australia

## Abstract

Over the past few decades, the field of p-type dye-sensitized solar cell (p-DSSC) devices has undergone tremendous advances, in which Cu-based delafossite nanocrystal is of prime interest. This paper presents an augment of about 87% improvement in photocurrent observed in a particular configuration of organic dye P1 sensitized CuCrO_2_ delafossite nanocrystal electrode coupled with organic redox shuttle, 1-methy-1H- tetrazole-5-thiolate and its disulfide dimer when Au nanoparticles (NPs, with diameter of about 20 nm) is added into the photocathode, achieving a power convert efficiency of 0.31% (measured under standard AM 1.5 G test conditions). Detailed investigation shows that the local electrical-magnetic field effect, induced by Au NPs among the mesoporous CuCrO_2_ film, can improve the charge injection efficiency at dye/semiconductor interface, which is responsible for the bulk of the gain in photocurrent.

Dye-sensitized solar cells (DSSC), one of the third generation solar cells, has attracted much attention because of high efficiency, low cost and easy fabrication^1^. It's the first to use a three-dimensional nanocrystalline junction for solar electricity production featured with separating light absorption from charge carrier transport. The adsorption of light in the DSSC occurs by dye molecules and the charge separation by electron injection from the dye to the nanocrystal (TiO_2_ for instance) at the semiconductor electrolyte interface. Optimizing kinetic efficiency in each step of this energy hierarchy is a principal goal of electrochemical energy research. Liquid-based DSSCs using porphyrins sensitizers currently attain 12.3% solar energy conversion efficiency and solid-state devices using perovskite light harvester with 15%, rendering it a credible alternative to other thin film photovoltaic cells^2^,^3^. The concept of the multi-junction or tandem solar cell was proposed with the goal to provide the possibility to absorb more photons, thereby leading to increase power conversion efficiency (PCE)^4^,^5^. Experimentally, this concept has been shown to be viable using two different sensitizers^5^, which can be matched to absorb the high energy photons on one electrode and low energy photons on the other, thereby utilizing the full solar spectrum^6^. Theoretically, the overall PCE of tandem DSSCs can reach above 40%^7^, superior to their single components. But presently, the efficiency of tandem DSSC devices is limited by the lower efficiency of p-type devices (the maximum reported value being about 1.3%). For this reason, there is a strong need for an improvement of the photovoltaic performance of p-type DSSCs. The operating principle of a p-type DSSC is shown by the schematic diagram in Figure 1. There are two key issues to address in order to increase the relatively low efficiencies found in p-type DSSC. One basic problem is a poor light harvesting found in p-type devices, resulting from the use of a thin nanocrystalline film (i.e., ~2 μm). The other impediment to producing efficient p-type DSSC devices is the low carrier mobility in the p-type semiconductor and mismatch energy level with sensitizers found at the dye-sensitized heterojunction (see Figure 1). One of the most recent achievements of p-type DSSCs research was the utilization of efficient sensitizing dyes^5^. Sun *et al* reported a series of organic donor-π-acceptor structured dyes for p-type DSSC having the triphyenylamine moieties as the electron donor, malononitrile moieties as the electron acceptor, and a thiophenes or other units as the conjugated chain, showing promising PCEs in the range of 0.1 ~ 0.2%^8^,^9^. Further improvements have been observed including an implantation of p-type semiconductor with high carrier mobility and suitable band energy level^10^,^11^,^12^, or new redox shuttles with high transparency and the steric bulk^13^,^14^. ABO_2_ oxides (A = Cu, Ag, B = transition metal element) bearing the delafossite structures have attracted a number of attractions as a triangular lattice antiferromagnet prototype with interesting properties such as transparent conductivity^15^ and multiferroicity^16^,^17^. Among recently studied compounds, the CuCrO_2_ delafossite is of prime interest due to its higher transparence, higher carrier mobility and more positive flat band in comparison with NiO^11^,^14^.

Recently, noble metal nanoparticles (NPs) such as Au and Ag with surface plasmon resonance (SPR) property constitute a large ongoing research field^18^, that have brought these nanostructures to the forefront of nanotechnology research directed towards applications in optoelectronics, including DSSCs^19^. The oscillation of conduction electrons in resonance with incident light promotes the electromagnetic field localized in the vicinity of plasmon resonance site, which has been verified to enhance optical absorption. Therefore the augmented photo-to-electron conversion efficiency could be attributed to the consequence of enhancement of optical absorption when Au NPs were added into DSSCs^20^,^21^. However, Zhang *et al* argued that the enhanced photocurrent was obtained from unexpected mechanisms of reducing exciton binding energy with help of Au@SiO_2_ NPs, rather than the enhancement of optical absorption^22^.

In this communication, we revisited CuCrO_2_^11^,^15^ and an organic sensitizer P1 (see Figure 1)^8^,^9^ in combination with a disulfide/thiolate redox shuttle (see Figure 1), presenting an impressive solar-to-electricity conversion efficiency of 0.31% under AM 1.5 G (air mass 1.5 global) (100 mW cm^−2^) when Au NPs were added into the photocathode. The magnetic field effect (MFE) offers a possible solution to show that the near field induced by Au NPs has an effect on photocurrent. In addition, the analysis supported that the near field promotes more charge injection at dye/semiconductor interface. This result is in agreement with earlier reported phenomena that the external electrical and magnetic fields have an effect on electron transfer reaction in various electrochemical systems, including electroluminescent diodes, fuel cells, organic solar cells, and n-type DSSCs^23^,^24^,^25^,^26^.

## Results

### CuCrO_2_ film characterization

**Figure 2:**
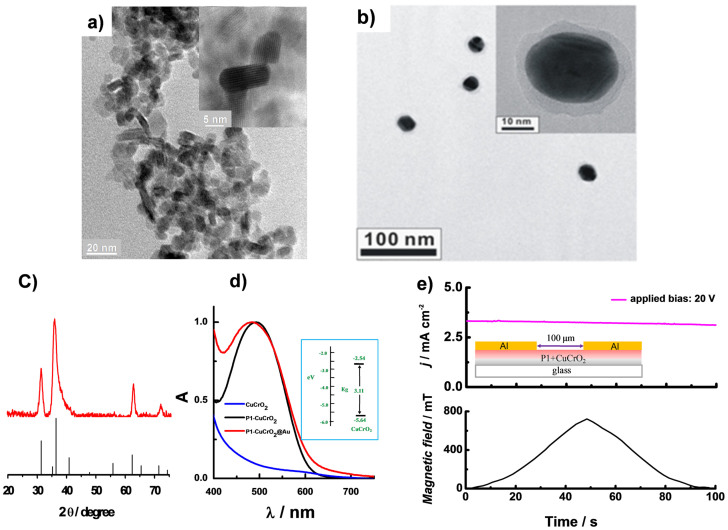
CuCrO_2_ film characterization. (a) TEM image of the synthesized CuCrO_2_ nanocrystals. Inset: High resolution TEM image. (b) TEM image of Au @SiO_2_ nanoparticles, inset: High resolution TEM image, (c) XRD pattern for CuCrO_2_ nanocrystalline mesoporous film. (d) Absorption spectra of CuCrO_2_ or CuCrO_2_@Au (CuCrO_2_ mixed with Au@SiO_2_) semiconductor film with and without sensitizer P1. The sample of CuCrO_2_ is presented for comparison purpose. Inset: schematic illustration of band gap of CuCrO_2_. (e) Current-voltage characteristic for in-plane devices containing CuCrO_2_ sensitized with P1 in a magnetic field. Inset: Schematic illustrate of the device structure. Film thickness: 3 μm, channel length: 100 μm.

Figure 2a presents the TEM images of ultrasmall CuCrO_2_ delafossite nanocrystals prepared by a hydrothermal method. The inset shows the high resolution image of CuCrO_2_ nanocrystals with a typical size of about 15 × 5 nm^2^ 11,15. The Au@SiO_2_ NPs were prepared by a standard chemical reduction synthesis method^27^. A representative TEM image of Au@SiO_2_ NPs is shown in Figure 2b. The Au NPs of about 20 nm diameter are coated by an approximate 3 nm SiO_2_ shell, showing an absorption peak at ~550 nm in water due to collective oscillation of electrons in nanoparticles (see Figure S1). Figure 2c presents the XRD phase analysis of CuCrO_2_, which confirms the delafossite crystalline structure with Cu^+^ in the lattice. The nanocrystal is predominantly c-axis oriented, with a very minor additional Bragg peak at the (101) orientation. Most traces of the Cu_2_O impurity phase were eliminated. Figure 2d presents the UV-visible spectrum of P1-sensitized CuCrO_2_ or CuCrO_2_ mixed with Au@SiO_2_ NPs (coded CuCrO_2_@Au) films. A slightly increased absorbance spectrum was observed in the wavelength range from 550 nm to 750 nm. The absorption peak due to the P1 dye was blue-shifted (from 495 nm to 482 nm) and the half width of absorption peak was broadened when Au NPs were mixed with CuCrO_2_. It was observed that the transparency of a 1.8 μm thick CuCrO_2_ or CuCrO_2_@Au film samples was 60% in the whole visible range. The inset of Figure 2d shows the estimated optical bandgap of CuCrO_2_ film, being about 3.11 eV.

### Photovoltaic performance

**Figure 3:**
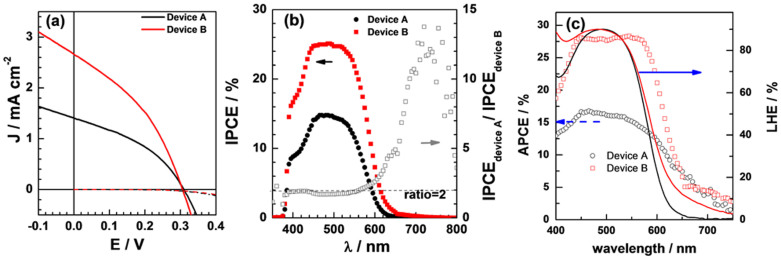
Photovoltaic performance of p-type DSSC based on CuCrO_2_ film. (a) Photocurrent density-voltage (J–V) and dark current characteristics of p-DSSCs using CuCrO_2_ (black curve, device A or CuCrO_2_@Au (red curve, device B) as the photocathode (1.8 μm film). The electrolyte composition of electrolyte in devices was 0.3 M T_2_ and 0.9 M T^−^ in acetonitrile-ethylene carbonate (7:3 volume ratio). Dotted lines correspond to the dark-current measurements. (b) Photo-current action spectra of p-type DSSCs based device A and device B. The incident photon to current conversion efficiency (left coordinate) and the ratio of IPCE _device B_/IPCE _device A_ (right coordinate) are plotted as a function of excitation wavelength. (c) Light-harvesting efficiency (solid line) and absorbed photon-to-electron conversion efficiency (dotted line) calculated with IPCE and the light harvesting efficiency.

Figure 3a presents the photocurrent density-voltage (J-V) and dark current characteristics of DSSCs using CuCrO_2_ (black curve, device A) or CuCrO_2_@Au (red curve, device B) as the photocathode (1.8 μm film thickness). The photovoltaic parameters, open circuit voltage (V_oc_), fill factor (FF), short circuit current density (J_sc_) and PCE of p-type DSSC devices under standard AM 1.5 G illumination at 100 mW cm^−2^ are tabulated in Table 1. Device A exhibited a V_oc_ of 0.309 V, a J_sc_ of 1.43 mA cm^−2^, and a FF of 0.38, giving an overall PCE of 0.17%. It is worthy to note the photocurrent line and the dark current line are crossing each other. The current-voltage characteristics for an in-plane file containing P1-sensitized CuCrO_2_ or bare CuCrO_2_ measured in dark and illumination are presented in Figure S2. A largely increased current for P1-sensitized CuCrO_2_ under illumination clearly demonstrated the photo-induced charge transfer process. An increasing hole density gives rise to CuCrO_2_ film conductivity and charge carrier mobility, which can be contributed to the cross of photocurrent and dark current. The larger current-voltage characteristic slope value of CuCrO_2_ film sensitized by P1 under illumination comparing to those in the dark implied that the enhanced conductivity only occurs when the photo-generated charge is sustained in CuCrO_2_ film^28^.

### Magnetic field effect

**Figure 4:**
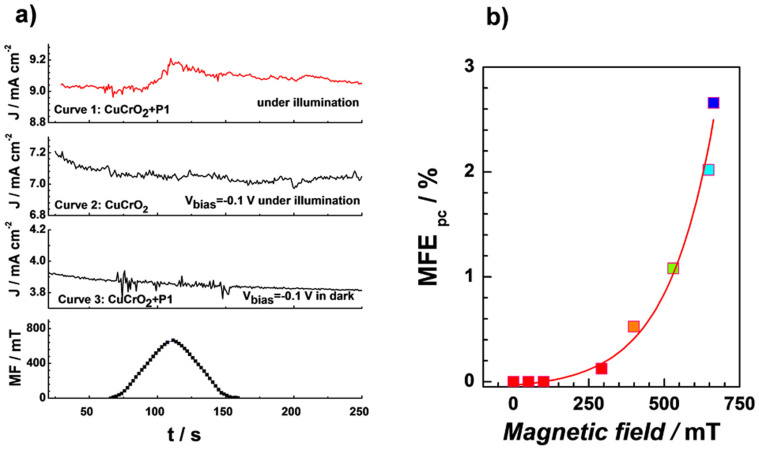
Magnetic field effect on p-type DSSC based on CuCrO_2_. (a) The magnetic field effect on photocurrent (MFE_PC_) (top three curves) and variation of magnetic field (bottom curve) are plotted as a function of time. The magnetic field effect on photocurrent response (top) of device using P1 sensitized CuCrO_2_ film (curve 1, red curve), CuCrO_2_ film (curve 2, black curve) measured at a bias of −0.1 V under illumination (about 2% sun light intensity), and P1 sensitized CuCrO_2_ film at a bias of −0.1 V in dark (curve 3, black curve), respectively. (b) The change of MFE_PC_ as a function of magnetic field intensities.

Magnetic field effects (MFE) measurements have been carried out with a home-made experimental setup by monitoring the device photocurrent (MFE_PC_)^29^. The current response of an in-plane film containing CuCrO_2_ sensitized with P1 in a magnetic field was first studied. The result in Figure 2e indicates that the magnetic field has negligible effect on the P1 sensitized CuCrO_2_ semiconductor film conductivity at room temperature when there is no redox shuttles. Figure 4a presents the photocurrent response (top) of a complete p-DSSC device using CuCrO_2_/P1 photocathode (red curve, curve 1) and device with bare CuCrO_2_ photocathode photocathode (black curve, curve 2) in combination with T_2_/T^−^ electrolyte at a bias of −0.1 V in a magnetic field (bottom) under illumination as a function of time. For comparison purpose, the MFE_PC_ measurement in the dark for the P1 based device was performed and the results were shown in Figure 4a (curve 3). Clearly, for a complete device under ~2% sun illumination condition the MFE_PC_ simultaneously increased with magnetic field intensity. A negligible change in the current signal was observed in curve 2 (without sensitizer) or curve 3 (in dark). Because of the instrument limitation, the magnetic field intensity could only reach its maximum value at ~700 mT, giving a MFE_PC_ improvement of ~3% (Figure 4b).

Since the photocurrent depends on the light harvesting capability, the absorbance of P1 sensitized CuCrO_2_ film in an increasing magnetic field was recorded as presented in Figure S3. The result showed that MFE had no effect on the absorption spectrum. Earlier studies have demonstrated that the magnetic field can increase photoconduction in terms of a hyperfine scale magnetic-field-dependent mixing of singlet and triplet electron-hole pair states, in which the concentration of singlet excitons can be modulated. The contribution of excitonic injection of holes to the drive current has been reported in Alq3-based electroluminescent diodes^25^. Therefore, based on the observation in Figure 2e and Figure 4 as well as Figure S3, we suggest that the enhanced MFE_PC_ can be due to an improvement in the charge injection efficiency at P1/CuCrO_2_ interface.

### Near field effect induced by Au NPs

As discussed above, the MFE_PC_ was observed in the p-type DSSC based on P1 sensitized CuCrO_2_ films in combination with T^−^/T_2_ electrolyte. It is reasonable to argue that photocurrent can be greatly enhanced if there is a magnetic field generated by a localized SPR. In order to verify this idea, silica-covered Au NPs (with the diameter of 20 nm) were synthesized and added into CuCrO_2_ film for SPR application in p-type DSSC. The Au NPs were mixed with CuCrO_2_ (Au:CuCrO_2_ 0.1% w/w) during the screen-printing paste preparation and the resulted films were sintered at 550°C in Ar atmosphere to promote a well-junction between Au NPs and CuCrO_2_ nanocrystals. Under the same condition for preparation of DSSC devices, changing the photocathode materials from CuCrO_2_ to CuCrO_2_@Au (the film thickness, the dye-loading and the rodox species concentration remain unchanged) in device B improved photocurrent by 87% to 2.68 mA cm^−2^, giving an overall PCE of 0.31% (Table 1).

Figure 3b presents the incident photon to current conversion efficiency (IPCE) as a function of the light excitation wavelength. The IPCEs below 390 nm excitation are deteriorated due to a sheet of anti-reflecting UV cut-off film attached to DSSCs. The features of the spectral response of the photocurrent closely match the adsorption spectrum of the P1 dye onto the CuCrO_2_ film (referring to Figure 2e). The IPCE of device A reaches its highest values of about 15% at about 470 nm, then drops rapidly above 530 nm. For device B, the IPCE increases largely from 440 nm to 530 nm, reaching over 25%. From the overlapped integral of this curve and the standard global AM 1.5 G solar emission spectrum, a short-circuit photocurrent density of 2.70 mA cm^−2^ is obtained, which is in excellent agreement with the measured photocurrent (J_sc_ = 2.68 mA cm^−2^), providing that the spectral mismatch between solar simulator and the standard AM 1.5 G solar emission is negligibly small. The ratio of IPCE _device B_/IPCE _device A_ was evaluated to be about 2 in the range from 400 nm to 580 nm (Figure 3b, right ordinate), evidently ascribing the augmented photocurrent. Considering the optical adsorption of the dye-sensitized films and IPCE spectra of the corresponding devices, the increased photocurrent in device B should not be caused by those spectrogram response after 600 nm though the ratio of IPCE _device B_/IPCE _device A_ increases largely in this wavelength range.

### Light harvesting property

Figure 3c presents the light harvesting efficiency (LHE) for the dye-sensitized nanocrystalline films examined by their UV-visible absorption spectra. As shown in Figure 3c (right coordinate), LHE is slightly increased for wavelength range from 550 nm to 800 nm due to the addition of Au@SiO_2_ NPs into the CuCrO_2_ film. As discussed above, the J_sc_ of device B had 87% enhancement while the V_oc_ values (Table 1) were nearly the same for both devices. We reasoned that the expected gain in J_sc_ could be caused by an in-situ electromagnetic field produced by Au NPs, due to the resonant collective oscillation of free electrons in noble metal nanostructures driven by light.

### Photovoltage transient decay measurement

**Figure 5:**
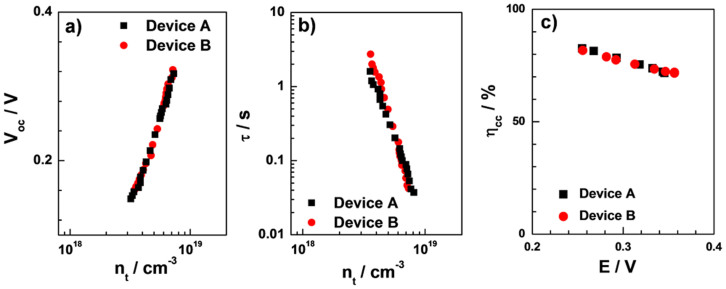
Transient decay measurements on p-type DSSC device based on CuCrO_2_. (a) Open circuit voltage, and (b) apparent charge lifetime as a function of injected hole density, (c) the charge collection efficiency as a function of potential.

The interfacial charge recombination kinetics and diffusion kinetics of injected holes were investigated by photovoltage transient decay experiments and charge extraction measurements. Figure 5a exhibits the V_oc_ as a function of the extracted charge density, n_t_ (where *n_t_* = Δ*Q*/(*A_r_d*/(1 − *p*)), Δ*Q* equals to the extracted charge, *A*_r_ being the real interface area of the photocathode, *d* being the film thickness of the photocathode, and *p* being the porosity of the cathode, respectively). The charge density was chosen as the abscissa in order to compare the V_oc_ values obtained from the two photocathodes at the same hole concentration in the dye-sensitized mesoporous films. All date points from both devices fall on the same straight line, suggesting that the addition of Au NPs to the CuCrO_2_ nanocrystals has negligible effect on the CuCrO_2_ valence band edge. Figure 5b shows the apparent charge lifetime (τ) as a function of injected hole density, allowing a comparison of the recombination rates for the different photocathodes at equal hole concentration in the films. The hole lifetime decreases with increasing the extracted charge density due to the higher hole density in the CuCrO_2_ layer and a larger driving force for the charge recombination. Figure 5c shows the charge collection efficiency (*η*_cc_, 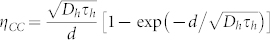, where τ_h_, D_h_, and *d* are the hole lifetime, hole diffusion coefficient and the film thickness, respectively) at the back TCO (FTO) as a function of open-circuit potential (*i.e.*, under different light intensity irradiation). Both devices give similar τ and *η*_cc_ values, clarifying that addition of Au NPs into CuCrO_2_ film has no effect on the interfacial charge recombination and the charge collection efficiency.

## Discussion

In this communication, it is the first time that an enhanced photocurrent due to the magnetic field effect was observed for DSSC devices endowed with p-type delafossite CuCrO_2_ and P1 dye in combination with organic thiolate-based electrolyte. The positive magnetic field effect on photocurrent of p-type DSSC based on CuCrO_2_ photocathode can be related to the tunning electron-hole pair dissociation in exciting singlet state dye^18^,^25^,^30^,^31^,^32^. Previously investigations suggested that an increase in photocurrent of DSSC devices associated with Au or other metal NPs was mainly attributed to the SPR effect^33^,^34^,^35^,^36^,^37^,^38^. In a majority of cases the evidence includes an increase in optical cross sectional areas of absorbers, thus a corresponding enhancement in PCE. Kamat *et al* also argued that the SPR effects gave rise to the photocurrent of DSSC while the charging effects of metal NPs leaded to an increase in photovoltage^31^. However, in this study we found that the addition of Au NPs into the CuCrO_2_ film brought negligible influence on the photon adsorption ability of the film and the interfacial recombination kinetic dynamics as well as the electronic energy levels of the semiconductor. Those parameters are related to the light-harvesting efficiency and the charge collection efficiency. It is well known that the IPCE spectra are determined by the sensitizer light-harvesting efficiency, the quantum yield for hole injection from the excited state of the dyes to the valence band of the semiconductor, and the collection efficiency of the hole at the back contact. Therefore, the IPCE gain observed in this study could be expected from an increase of the charge injection from the excited P1 dyes into CuCrO_2_ valence band.

In order to investigate the nature of the charge injection efficiency enhancements at visible wavelengths, the internal quantum efficiency, or absorbed photon conversion efficiency (APCE) of the tested devices were determined. These efficiencies were calculated through the following equations: 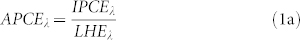


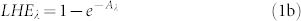
where *A*_λ_ is the absorbance of a component in the layered structure, corrected for the substrates and the electrolyte, *LHE*_λ_ is the light harvesting efficiency. The APCE proves a precise representation of the intrinsic light conversion efficiency. The LHE and APCE values determined by equation 1a and 1b are plotted in Figure 3c. The device B shows a broader and higher APCE response above 480 nm than that of device A. At wavelength of 500 nm (close to the adsorption peak of P1 sensitizer, see Figure 2d), the APCEs values are about 16% and 28% for devices A and B (Figure 3c) respectively. This observation confirms the proposed benefits of the heterojunction architecture as discussed above: stronger APCE response at visible wavelengths suggesting an enhancement of charge separation. This result also indicates that the Au NPs-doped CuCrO_2_ can improve the absorbed photon-to-current conversion efficiency through an increased dissociation of excitons.

**Figure 6:**
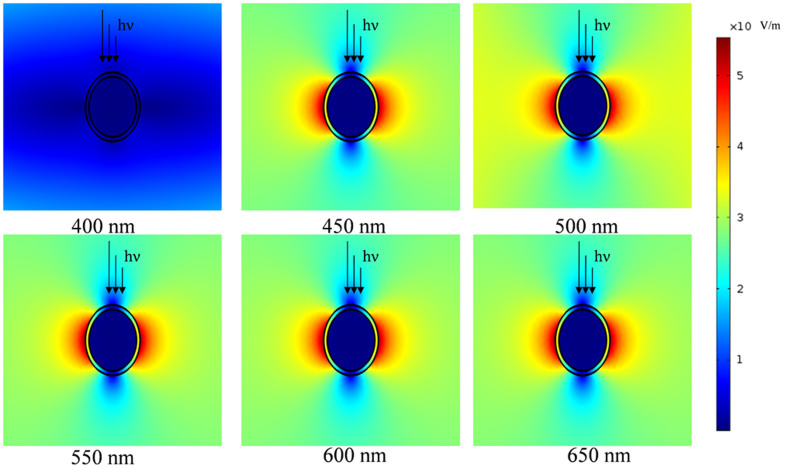
The simulated local electromagnetic field based on a 2-D model. The simulation of electromagnetic field induced by NPs was carried out based on a two-dimensional model within the finite element method supported by COMSOL Multiphysics software (CnTech). A periodic boundary with a unit cell of 1 μm was used.

Several works have demonstrated that the electromagnetic field can be remarkably changed by the light polarization^39^. Theoretical and experimental results have disclosed the relation between the SPR and magnetic filed, showing that the excitation of surface plasmons can greatly enhance electromagnetic fields^40^,^41^,^42^. The effect of the light wavelength on the intensity of local electromagnetic field around Au@SiO_2_ surface was evaluated (see Figure 6). The simulation was carried out based on a two-dimensional model at the finite element method supported by COMSOL Multiphysics software (CnTech). Simulation results showed that a strong electromagnetic field (about 50 V m^−1^) could be generated near onto the surface of Au NPs for the incident irradiation in the range from 450 to 650 nm, but decreased quickly with increasing distance from the surface of Au@SiO_2_ NPs.

The dye molecules can collect the light energy and transfer their singlet excitation (functioning as an antenna) and, at the same time, dissipate the singlet and triplet energies (functioning as a self-quencher). For a series of changes among the singlet-excited and redox states having a singlet character in P1-sensitized CuCrO_2_ device, upon absorption of photon, electron is transferred to a higher singlet level. Then hole injection takes place to generate a charge-separated state having a singlet character on the P1/CuCrO_2_ boundary. Hole is transferred further into CuCrO_2_ to form a stable charge-separated state. A reverse electron transfer followed by charge recombination takes place to relax into the ground state. If considering the generation of the triplet-excited and radical-cation states both having a triplet character, singlet excitons can be partially converted into triplet excitons through intersystem crossing^26^. Both singlet and triplet excited states have different contributions to the charge dissociation owing to their different binding energies. A positive magnetic field effect was observed in small molecules, which was attributed to a change in the conversion rate between singlet and triplet prior to recombination^43^. In this study, the tripheneylanmine moiety of the P1 sensitizer presents an aromatic molecular structure. The internal splitting energy was assumed to be about 1–10 μeV^44^. This small internal splitting energy suggested that a magnetic field of 100 mT or a local electromagnetic field of 50 V m^−1^ could influence the intersystem crossing process and consequently change the singlet and triple ratios.

This study showed that the addition of Au NPs into a p-type oxide semiconductor delafossite CuCrO_2_ film used for dye-sensitized solar cells significantly increased the cell performance. The presence of 0.1 wt% Au NPs in a CuCrO_2_ based p-type DSSCs increased the PCE to 0.31%. This result should be extendable to other p-type delafossite materials of appreciate conductivity. It leads to the general importance of increasing charge injection in p-type DSSC, and their adverse effects on both J_sc_ and power conversion efficiency.

## Methods

### Synthesis of spherical Au@SiO_2_ nanoparticles

1 mL sodium citrate (10 mM) and 1 mL HAuCl_4_ (10 mM) were dissolved in 37 mL deionized water. Then, 1 mL NaBH_4_ (0.1 M) was added into the above solution. This produces Au seeds solution. Meanwhile, 50 mM CTABCl was added into 100 mL HAuCl_4_ (0.25 mM) aqueous solution, followed by 0.5 mL ascorbic acid (100 mM), 0.5 mL NaOH (100 mM) solution,and finally the 0.1 mL above-mentioned Au seeds solution. After 10 h, the mixture was dissolved into 5 mL NaOH (1 μM) aqueous solution again. Lastly, 10 μL TEOS was added into the solution and stirred for 10 h.

### Devices fabrication and characterization

The CuCrO_2_ film was sintered at 420°C for 40 min then 550°C for 40 min in Ar. After cooled down to about 80°C, the film electrodes were dipped into a 300 μM P1 solution in acetonitrileat room temperature for 16 hrs. After washed with acetonitrile and dried by air flow, the sensitized CuCrO_2_ electrodes were assembled with counter electrodes. The working electrodes and counter electrodes were separated by a 45 μm thick hot melt ring (Surlyn, Dupont) and sealed by heating. The internal space was filled with liquid electrolytes using a vacuum back filling system. The electrolyte for devices was 0.3 M T_2_ and 0.9 M T^−^ with the tetramethylammonium cation in the mixture of acetonitrile and propylene carbonate (volume ratio, 7:3).

A 450 W xenon light source solar simulator (Oriel, model 9119) with AM 1.5 G filter (Oriel, model 91192) was used to generate an irradiance of 100 mW cm^−2^ at the surface of the test cells. The current-voltage characteristics of the cells under these conditions were obtained by applying external potential bias to the cell and measuring the generated photocurrent with a Keithley model 2400 digital source meter (Keithley, USA). A similar data acquisition system was used to control the IPCE measurement. A white light bias (1% sunlight intensity) was applied onto the sample during the IPCE measurements with ac model (10 Hz).

### Photovoltage/photocurrent transient decay and charge extraction measurements

For the photovoltage transient decay measurement, a white light bias was generated by an array of diodes. Red light pulse diodes (0.05 s square pulse width, 100 ns rise and fall time) controlled by a fast solid-state switch were used as the perturbation source. The voltage dynamics were recorded on a PC-interfaced Keithley 2602A source meter with a 100 μs response time. The perturbation light source was set to a suitably low level in order for the voltage decay kinetics to be monoexponential. By varying the white light bias intensity, the recombination rate constant and the hole diffusion rate constant could be estimated over a range of applied biases, which were used to evaluate the charge collection efficiency (η_cc_). Before the LEDs switched to the next light intensity, a charge extraction routine was executed to measure the electron density in the film. In the charge extraction techniques, the LED illumination source was turned off in <1 μs, while simultaneously, the cell was switched from open to short circuit, in which a resistance is series connected to the device. The resistance is adjusted close to the corresponding value at maximum power point of the device. The resulting current, as the cell returns to V = 0 and J = 0, was integrated to give a direct measurement of the excess charge in the film at that V_oc_, which is the minimum level of charge in the semiconducting photocathode.

### Magnetic field effect measurements

To define the effect of external magnetic fields, the test cells were placed between the pole pieces of an electromagnet. The surface of sample was parallel to the magnetic field. A simulated sunlight (with of about 1% of full sun intensity) was introduced by optical fiber (Newport, 77632). The generated photocurrent dates were recorded on a PC-interfaced Keithley 2400 source meter.

## Author Contributions

M.W., Y.S., W.C., S.Y. and B.H. contributed to the conception and design of the experiment, analysis of the data and writing the manuscript with assistance of X.X. and Y.C., J.C., J.H., J.Z., Y.Z., L.L., G.A., Z.W., D.X. and Z.W. carried out synthesis of materials, preparation of the devices, device performance measurements and writing experimental part in the manuscript.

## Supplementary Material

Supplementary Information SI-revised

## Figures and Tables

**Figure 1:**
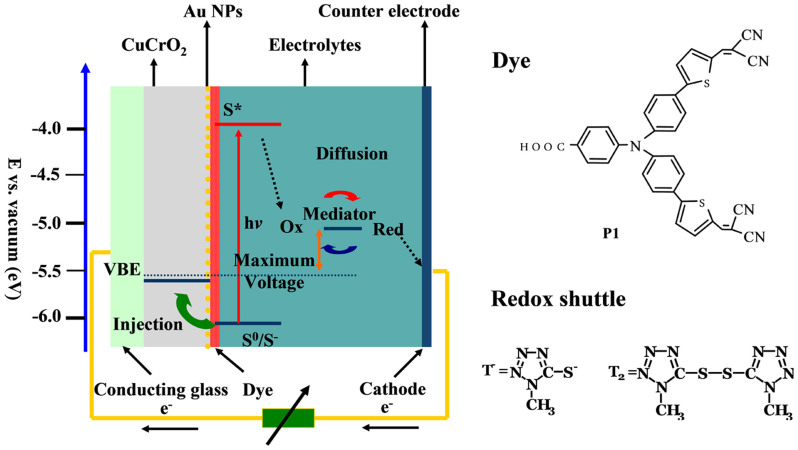
Energy band diagram of a P1-sensitzied CuCrO_2_ solar cell. The molecular structures of P1 dye and redox shuttle are presented.

**Table 1:** Photovoltaic parameters of devices basing on CuCrO_2_ (device A) and CuCrO_2_@Au (CuCrO_2_ mixed with Au@SiO_2_ NPs, device B) photocathodes with 1.8 μm thickness under AM 1.5 illumination

	V_oc_ [V]	J_sc_ [mA cm^−2^]	FF	PCE [%]
Device A	0.309	1.43	0.38	0.17
Device B	0.305	2.68	0.38	0.31
