# Supplementary material for: Near Field Enhanced Photocurrent Generation in P-type Dye-Sensitized Solar Cells
Source: Sci Rep. 2014 Feb 4;4:3961. doi: 10.1038/srep03961 (PMC3912483; doi:10.1038/srep03961)
Supplement: Supplementary Information — SI-revised [file srep03961-s1.doc]

Supporting information

Journal: Scientific Reports

Near Field Enhanced Photocurrent Generation in P-type Dye-Sensitized Solar Cells

Xiaobao Xu^1^, Jin Cui^1^, Junbo Han^2^, Junpei Zhang^2^, Yibo Zhang^1^, Lin Luan^1^, Getachew Alemu^1^, Zhong Wang^1^, Yan Shen^*1^, Dehua Xiong^1^, Wei Chen^*1^, Zhanhua Wei^3^, Shihe Yang^3^, Bin Hu^1,4^, Yibing Cheng^1,5^, Mingkui Wang^*1^

^1^ Michael Grätzel Center for Mesoscopic Solar Cells, Wuhan National Laboratory for Optoelectronics, School of Optical and Electronic Information, Huazhong University of Science and Technology, 1037 Luoyu Road, 430074 Wuhan, P. R. China

^2^ Wuhan National High Magnetic Field Center, School of Physics, Huazhong University of Science and Technology, 1037 Luoyu Road, 430074 Wuhan, P. R. China

^3^ Department of Chemistry, The Hong Kong University of Science and Technology, Clear Water Bay, Kowloon, Hong Kong, China

^4^ Department of Materials Science and Engineering, University of Tennessee, Knoxville, TN 37996-2100

^5^ Department of Materials Engineering, Monash University, Melbourne, Victoria, 3800, Australia

**Figure S1** UV-visible spectroscopy measurement of Au@SiO_2_ in water.

**Figure S2.** Current-voltage characteristic for in-plane devices containing CuCrO_2_ or CuCrO_2_ sensitized with P1 under dark or illumination. Inset: Schematic illustrate of the device structure. Film thickness: 3 μm, channel length: 100 μm.

**Figure S3.** Absorption spectrum of P1 sensitized CuCrO_2_ in transient magnetic field with different strength.
